# Real-World Validation of an AI-Based Opportunistic Osteoporosis Risk Stratification Tool Using Chest Radiographs in a Male-Predominant Cohort

**DOI:** 10.3390/healthcare14193200

**Published:** 2026-09-28

**Authors:** Kao-Chang Tu, Sheng-Chou Wu, Han-Ting Shih, Cheng-Hung Lee, Kun-Hui Chen, Chia-Hsien Hsu

**Affiliations:** 1 Department of Orthopaedics, Taichung Veterans General Hospital, Taichung 40705, Taiwan; 2 Doctoral Program in Tissue Engineering and Regenerative Medicine, National Chung Hsing University, Taichung 402202, Taiwan; 3 Department of Industrial Engineering and Enterprise Information, Tunghai University, Taichung 407224, Taiwan; 4 Department of Post-Baccalaureate Medicine, College of Medicine, National Chung Hsing University, Taichung 402202, Taiwan; 5 Department of Computer Science and Information Engineering, Providence University, Taichung 43301, Taiwan; 6 Institute of Biomedical Engineering and Nanomedicine, National Health Research Institutes, Zhunan, Miaoli County 35053, Taiwan; 7 Institute of NanoEngineering and MicroSystems, National Tsing Hua University, Hsinchu 30013, Taiwan

**Keywords:** osteoporosis, artificial intelligence, chest radiograph, opportunistic screening, bone mineral density

## Abstract

**Background:** Osteoporosis is a major public health concern and a leading cause of fragility fractures, yet screening with dual-energy X-ray absorptiometry (DXA) remains limited by accessibility and utilization. Opportunistic artificial intelligence (AI)-based analysis of routinely acquired chest radiographs (CXRs) may facilitate osteoporosis risk stratification. This study aimed to independently validate a regulatory-approved deep learning model (VeriOsteo™ OP) in a real-world, male-predominant cohort. **Methods:** We retrospectively analyzed 2336 patients who underwent both CXR and DXA at a medical center in 2023, including 1688 males (72.3%) and 648 females (27.7%). Osteoporosis was defined as a DXA T-score ≤ −2.5. AI performance was evaluated using sensitivity, specificity, positive predictive value (PPV), negative predictive value (NPV), accuracy, and area under the receiver operating characteristic curve (AUC), with sex-specific subgroup analyses. **Results:** The AI model achieved an accuracy of 76.97%, sensitivity of 69.67%, specificity of 84.64%, PPV of 82.66%, and NPV of 72.65%. The overall AUC was 0.854 (95% CI, 0.839–0.868). The AUC was 0.825 (95% CI, 0.794–0.854) in males and 0.849 (95% CI, 0.831–0.866) in females. PPV was lower in males than in females (60.00% vs. 83.39%), while other performance measures were broadly comparable. The optimal AI cutoff also differed between males (≤−1.9) and females (≤−2.5). **Conclusions:** The AI model demonstrated good discrimination for opportunistic osteoporosis risk stratification using CXRs in a real-world cohort. Its moderate sensitivity and lower PPV among males highlight potential sex-related differences in AI-assisted osteoporosis assessment. The model may be most appropriately used as an adjunctive risk-stratification and DXA-prioritization tool to identify individuals who may benefit from subsequent DXA evaluation rather than a standalone diagnostic test.

## 1. Background

Osteoporosis, a common chronic metabolic bone disease, is defined by reduced bone mineral density (BMD) and microarchitectural degradation of bone tissue. These changes result in porous bones and a heightened susceptibility to fractures [1]. The condition is frequently termed the “silent disease” because it remains largely asymptomatic prior to the occurrence of a fracture [2]. The burden of osteoporosis-related fractures, especially vertebral and hip fractures, on healthcare systems is immense, contributing to significant costs related to hospitalization, long-term care, and disability [3]. The escalating prevalence of osteoporosis, driven by a globally aging demographic, has established it as a primary public health concern [4]. In Taiwan, for instance, the Nutrition and Health Survey in Taiwan (NAHSIT 2004–2008) revealed a prevalence of 22.57% in men and 41.17% in women over the age of 50 [5]. Nevertheless, screening for this condition remains inadequate, a situation attributable to insufficient public awareness and barriers to healthcare access.

The early identification of individuals with osteoporosis is paramount for fracture risk assessment and the timely implementation of preventive interventions. The current gold standard for diagnosing osteoporosis is dual-energy X-ray absorptiometry (DXA), which measures bone mineral density (BMD) at critical sites such as the lumbar spine and hip [6]. Nevertheless, its widespread application for population screening and therapeutic monitoring is hampered by limited accessibility and considerable cost [7]. Although quantitative ultrasound (QUS) presents a more affordable and portable alternative [8], its diagnostic accuracy is often inferior. A study in a Taiwanese cohort, for example, reported that when compared with DXA, QUS screening for lumbar osteoporosis yielded a sensitivity of only 67.2% and a specificity of 64.9% [9,10]. Consequently, these constraints contribute to a substantial underdiagnosis of the condition, underscoring the urgent need for more effective and accessible screening strategies.

The emergence of artificial intelligence (AI) presents a valuable opportunity to revolutionize osteoporosis screening through “opportunistic screening”—a paradigm that leverages existing medical images to identify at-risk individuals [11,12]. This approach can significantly boost detection rates without imposing extra radiation, costs, or time on patients. While deep learning models have been successfully applied to radiographs of the hand, lumbar spine, and pelvis for this purpose [13,14,15], these images are not universally performed. This limitation can undermine the core principle of opportunistic screening if additional procedures are required. To overcome this, our study strategically employs chest radiographs (CXRs), which are among the most frequently conducted imaging studies globally and are a staple of routine health examinations, particularly in many Asian populations [16].

Building on this premise, we developed VeriOsteo™ OP (Acer Medical Inc., New Taipei City, Taiwan), an AI-powered software designed for opportunistic osteoporosis screening from CXRs. The tool uses a deep learning algorithm to analyze the thoracolumbar junction (T12-L1)—a region highly susceptible to osteoporotic compression fractures [17]—and was trained against the gold-standard reference: dual-energy X-ray absorptiometry (DXA)-derived bone mineral density. However, a critical gap persists in the literature: a scarcity of AI diagnostic tools that have undergone the rigors of pivotal clinical trials and secured regulatory approval. This crucial step is what separates a research algorithm from a clinically viable medical device. While the VeriOsteo™ OP model demonstrated high efficacy in its pivotal study [17], that cohort was predominantly female (82%). However, the generalizability of such deep learning models to male-predominant populations remains largely under-explored. Rather than developing a novel algorithm, this study conducted an independent validation of VeriOsteo™ OP using a real-world cohort from a Veterans General Hospital (72.3% male). Serving as a critical “stress test” under a substantially different demographic distribution, our objective was to assess model robustness against demographic domain shift and clarify the clinical utility of AI-based opportunistic osteoporosis screening across biological sexes in routine practice.

## 2. Materials and Methods

### 2.1. Study Design and Population

This retrospective validation study was conducted at Taichung Veterans General Hospital, a single medical center in central Taiwan. The study protocol was approved by the Institutional Review Board (IRB) of Taichung Veterans General Hospital, and the requirement for informed consent was waived due to the anonymized, retrospective nature of the analysis.

We initially identified 18,500 individuals who underwent a posteroanterior (PA) chest radiograph (CXR) between 1 January 2023 and 31 December 2023 by searching the hospital’s Picture Archiving and Communication System (PACS). From this initial cohort, patients were considered eligible if they had both an eligible CXR and a dual-energy X-ray absorptiometry (DXA) examination performed within a 12-month interval. The inclusion criteria were: (1) age ≥ 50 years; (2) both CXR and DXA examinations performed within a 12-month interval; and (3) CXR images of adequate diagnostic quality for analysis. Patients were excluded if they had significant spinal hardware, severe scoliosis that could interfere with image analysis, or incomplete demographic or DXA data.

For determination of the DXA-based ground truth, lumbar vertebrae were excluded at the vertebral level when the T-score difference between adjacent vertebrae exceeded 1.0 SD, consistent with ISCD recommendations, suggesting potential artifact or structural abnormality. If lumbar spine DXA data were unavailable or excluded according to these predefined criteria, the available hip DXA data were used for osteoporosis classification when applicable. Patients with non-interpretable lumbar spine DXA measurements remained eligible for inclusion in the final analytical cohort when an appropriate alternative DXA reference measurement was available. The patient selection process is summarized in Figure 1.

This study utilized a completely independent dataset collected between 1 January 2023, and 31 December 2023. There is no temporal or patient overlap with the dataset used in the previous pivotal study [17], which analyzed data from 2016 to 2020.

The clinical indications for CXR were varied and commonly included routine health examinations, pre-operative assessments, and evaluation of respiratory symptoms. DXA examinations were performed based on clinical assessment and the presence of indications or risk factors for osteoporosis, according to local clinical practice and applicable national recommendations.

### 2.2. Data Collection and Reference Standard

For each patient, we collected baseline demographic data, including age, sex, height (cm), and weight (kg). Body Mass Index (BMI) was calculated as weight in kilograms divided by the square of height in meters (kg/m^2^).

The reference standard for osteoporosis diagnosis was the T-score obtained from the DXA scan (GE Lunar iDXA system revision 9 (Madison, WI, USA)), based on the World Health Organization (WHO) criteria. The final DXA T-score for each patient was defined as the lowest T-score measured from the lumbar spine (L1-L4), or femoral neck. According to WHO guidelines, patients were categorized as having osteoporosis if their DXA T-score was −2.5 or lower, and non-osteoporosis if the T-score was greater than −2.5.

### 2.3. AI Model Analysis (Index Test)

The index test was VeriOsteo™ OP (Version 1.00.3000; Acer Medical Inc., New Taipei City, Taiwan), a deep learning-based software as a medical device (SaMD). The software holds a Class II medical device license from the Taiwan Food and Drug Administration (TFDA; License No. [007973]).

The model is designed to analyze standard PA chest radiographs. It automatically identifies the thoracolumbar junction (T12-L1) as its region of interest and generates a predicted bone mineral density T-score (hereafter referred to as “CXR T-score”) based on the image features. The model was previously trained and validated against a large dataset with DXA-derived T-scores as the reference standard [17]. For this study, the CXR images corresponding to each patient’s DXA scan were processed by the AI model in a fully automated manner, with the operators blinded to the DXA results.

### 2.4. Statistical Analysis

Descriptive statistics were used to summarize the characteristics of the study population. Continuous variables (age, height, weight, BMI, and T-scores) were presented as mean and standard deviation (SD).

The primary analysis focused on evaluating the diagnostic accuracy of the AI model in identifying osteoporosis. Using the DXA T-score as the reference standard, we created a binary classification (osteoporosis vs. non-osteoporosis). The diagnostic performance of the CXR AI model was then assessed by calculating its sensitivity, specificity, positive predictive value (PPV), negative predictive value (NPV), and overall accuracy.

To evaluate the diagnostic performance of the AI model, the continuous CXR T-score was dichotomized to create a binary classification. Consistent with the WHO diagnostic criteria for the reference standard, a CXR T-score of −2.5 or lower was defined as a positive result (indicative of suspected osteoporosis), while a score greater than −2.5 was considered negative.

Using the DXA classification as the ground truth, a 2 × 2 contingency table was constructed. We then calculated the model’s sensitivity, specificity, positive predictive value (PPV), negative predictive value (NPV), and overall accuracy. Furthermore, a Receiver Operating Characteristic (ROC) curve was generated to assess the model’s overall discriminative ability across all possible thresholds, and the Area Under the Curve (AUC) was calculated. The 95% confidence intervals (CIs) for sensitivity, specificity, PPV, NPV, and accuracy were calculated using the Wilson score method. The 95% CI for the AUC was calculated using a nonparametric method.

The primary diagnostic performance analysis was performed using the prespecified CXR/AI T-score threshold of ≤−2.5. Sex-specific CXR/AI thresholds were subsequently derived using the Youden index as a secondary, exploratory analysis. For sex-stratified analyses, diagnostic performance metrics were calculated separately for male and female subgroups. Categorical diagnostic performance measures were compared between subgroups using the chi-square test or Fisher’s exact test, as appropriate. AUCs between male and female subgroups were compared using DeLong’s test.

All statistical analyses were performed using IBM SPSS Statistics (Version 26.0; IBM Corp., Armonk, NY, USA). A *p*-value of less than 0.05 was considered statistically significant.

## 3. Results

### 3.1. Patient Characteristics

During the study period, approximately 18,500 patients underwent chest radiography, of whom 2700 also underwent DXA. After applying the predefined eligibility and exclusion criteria, 2336 patients were included in the final analysis. Of these 2336 patients, lumbar spine DXA measurements were excluded at the vertebral level according to the predefined criteria in 207 patients. These patients remained in the final analytical cohort, and available hip DXA measurements were used for osteoporosis classification when applicable. The demographic and clinical characteristics of the study cohort are summarized in Table 1. The mean age of the patients was 69.9 ± 10.4 years. The majority of the participants were male (72.3%, 1688/2336). The male cohort showed a broad distribution of age, BMI, and DXA T-scores, reflecting the clinical heterogeneity of a real-world population. This substantial and clinically heterogeneous cohort provides an appropriate population for evaluating the performance of VeriOsteo™ OP in men.

The mean DXA T-score (the reference standard) was −2.4 ± 1.1, while the mean T-score specifically for the lumbar spine was −1.3 ± 1.8. The AI model generated a mean CXR T-score of −2.1 ± 1.0 for the cohort. The mean interval between CXR and DXA examinations was 126.4 ± 108.9 days, with a median of 102 days (range, 0–365 days).

### 3.2. Diagnostic Performance of the CXR AI Model

To evaluate the clinical utility of the CXR AI model, we assessed its ability to detect osteoporosis using the standard DXA T-score threshold of ≤−2.5. The performance of the model against the DXA reference standard is presented in Table 2.

The AI model demonstrated a sensitivity of 69.67% in identifying patients with osteoporosis. The specificity for correctly identifying non-osteoporotic patients was 84.64%. The overall accuracy of the model was 76.97%. The positive predictive value (PPV) was 82.66%, and the negative predictive value (NPV) was 72.65%. The AI-predicted T-scores were highly correlated with DXA T-scores (Pearson r = 0.732, *p* < 0.001) (Figure 2).

To further evaluate the impact of sex on model performance, a subgroup analysis was conducted (Table 3). For all subgroup analyses, osteoporosis was consistently defined as a DXA T-score of ≤−2.5. The optimal CXR/AI cutoff was independently determined for each sex subgroup using ROC curve analysis and the Youden index, and the corresponding diagnostic performance measures were calculated using the respective subgroup-specific CXR/AI cutoff. The model demonstrated superior discriminative ability in the female cohort, achieving an AUC of 0.849 (95% CI: 0.831–0.866), with a specificity of 79.09% and a positive predictive value (PPV) of 83.39%. In contrast, the performance in the male cohort was lower, with an AUC of 0.825 (95% CI: 0.794–0.854). Notably, the sensitivity was comparable between sexes (approximately 74–75%), whereas the PPV was numerically lower in males than in females (60.00% vs. 83.39%), likely reflecting the lower prevalence of osteoporosis and potential confounding factors such as degenerative changes in the male-predominant population (Figure 3). Because the sex-specific CXR/AI cutoffs were derived and evaluated within the same cohort as a secondary, post-hoc exploratory analysis (i.e., in-sample optimization), the corresponding sex-stratified performance estimates should be considered exploratory and require further validation in independent cohorts. These cutoffs do not alter the DXA-based definition of osteoporosis (T-score ≤ −2.5) and should not be interpreted as established clinical thresholds.

## 4. Discussion

In this independent real-world validation cohort, VeriOsteo™ OP demonstrated moderate-to-good overall discriminative performance for osteoporosis, with an AUC of 0.854 and a specificity of 84.64%. However, its performance was lower than that reported in the original pivotal validation, particularly in terms of sensitivity. Importantly, our cohort differed substantially from the previous validation population, with 72.3% of participants being male. These findings suggest that the clinical performance of an AI-based osteoporosis risk-stratification tool may be influenced by demographic differences between the development and deployment populations, highlighting the importance of external validation before broad clinical implementation.

Our model demonstrated a significant correlation between the predicted T-score from CXR and the observed T-score derived from DXA (Pearson’s r = 0.732, *p* < 0.001). This finding is positioned favorably within the context of existing literature. For instance, Sato et al. (2022) reported a correlation coefficient (R) of 0.63 for lumbar BMD prediction using chest X-rays combined with age and sex as multimodal inputs [18]. In comparison, the pivotal validation study of the VeriOsteo™ OP model by Tseng et al. (2024) reported a Pearson’s r of 0.88 [17], while our study yielded a Pearson’s r of 0.732 using radiographic image features alone. Given the differences in model architecture and input variables across these studies, these correlation coefficients should be interpreted as contextual rather than direct head-to-head performance comparisons. It should also be noted that Pearson’s correlation coefficient is sensitive to the distribution and range of T-scores within a given cohort. Therefore, cross-study comparisons of r values should be interpreted with caution, as differences in cohort characteristics and T-score distributions may contribute to variations in the observed correlations. Nevertheless, our findings support the ability of the model to extract features relevant to bone mineral density from routine chest radiographs. Of particular note, a recent investigation by Yoshida et al. (2025) revealed that incorporating bi-planar (frontal and lateral) CXR views as model input substantially enhances the accuracy of lumbar BMD estimation (R = 0.766) compared to using frontal views alone (R = 0.709) [19]. This suggests that the performance of our model could be further augmented through the future integration of multi-view radiographic data.

In terms of diagnostic performance for osteoporosis (defined as a DXA T-score ≤ −2.5), the AI model in our study yielded an overall accuracy of 76.97%, a sensitivity of 69.67%, a specificity of 84.64%, a positive predictive value (PPV) of 82.66%, and a negative predictive value (NPV) of 72.65%. While the sensitivity was moderate at 69.67%, the model exhibited robust specificity at 84.64%. A performance gap is evident when these metrics are benchmarked against those from the pivotal clinical validation of VeriOsteoTM OP by Tseng et al. (2024), which reported an overall accuracy of 89.0%, sensitivity of 88.7%, and specificity of 89.4% [17]. This observed disparity may be attributable to several factors:

Disparities in Cohort Characteristics and Domain Shift: The primary factor contributing to the decrease in AUC (0.854 in this study vs. 0.948 in the pivotal study [17]) is likely the demographic inversion between the datasets. The original model was trained on a population that was 78% female [17]. In contrast, our real-world cohort is 72.3% male. The lower performance observed in males may potentially be related to sex-specific differences in radiographic appearance and the greater prevalence of degenerative changes, such as osteophytes or aortic calcification, which could potentially affect the radiographic features available to the AI model. However, this explanation remains speculative and unverified in our current dataset, as these radiographic features were not systematically graded or quantitatively assessed. Our subgroup analysis (Table 3) demonstrated a difference in model performance between females (AUC 0.849) and males (AUC 0.825), but this finding does not establish a causal relationship between these radiographic features and model performance. This finding highlights a critical challenge in AI deployment: performance degradation due to domain shift in patient demographics, emphasizing the need for sex-specific model fine-tuning.

Type of Imaging Input: The original model from Tseng et al. (2024), which our study is based on, was developed primarily using posteroanterior (PA) view chest radiographs focusing on the T12-L1 region [17]. As previously discussed, research by Yoshida et al. (2025) has compellingly shown that incorporating both frontal and lateral CXR views enhances performance in BMD estimation and osteoporosis screening [19]. Consequently, the model’s reliance on a single radiographic projection may inherently limit its capacity to extract a more comprehensive set of features correlated with bone mineral density.

Trade-offs in the Context of Opportunistic Screening: For opportunistic risk stratification, high sensitivity may be prioritized to maximize the detection of individuals at risk, potentially at the expense of specificity. Our model’s sensitivity of 69.67% implies that a subset of osteoporotic patients would be missed, indicating that the model should not be used to exclude osteoporosis or replace DXA, particularly in patients with high clinical risk. Conversely, the specificity of 84.64% may help prioritize patients for confirmatory DXA assessment and potentially reduce unnecessary referrals compared with indiscriminate DXA testing.

Nevertheless, our study possesses several key strengths. First and foremost, we conducted an independent validation of a regulatory-approved AI model on a novel 2023 dataset. This approach substantially enhances the model’s external validity, confirming its generalizability and performance on unseen, real-world data. Secondly, the large scale of our validation cohort, comprising 2336 patients, provides robust evidentiary support for the model’s real-world applicability. Finally, this study highlights the profound practical utility of leveraging chest radiographs for osteoporosis risk stratification. Given the widespread availability of chest radiographs in routine clinical practice, their use for opportunistic osteoporosis screening may facilitate integration into existing clinical workflows without requiring additional imaging procedures or radiation exposure [20].

Nonetheless, this study has several limitations. First, the model showed moderate sensitivity (69.67%), indicating that some patients with osteoporosis may be missed. Therefore, the model should currently be used mainly for opportunistic risk stratification and DXA prioritization rather than as a standalone diagnostic tool.

Second, our validation cohort had a male predominance (72.3%), which differed from the female-majority population of the pivotal study. This difference may partly reflect differences in the clinical indications for DXA between women and men. Postmenopausal women are commonly evaluated based on age and menopausal status, whereas men may be referred for DXA because of additional clinical risk factors, previous fractures, or suspected secondary osteoporosis. This demographic difference may have contributed to differences in model performance. In addition, the relatively smaller number of female participants (648 vs. 1688 males) may have limited the statistical power and generalizability of the sex-stratified findings in women. As this was a single-center study conducted in a predominantly male population, the observed sex-related differences should therefore be interpreted cautiously and may not be directly generalizable to broader or more sex-balanced populations. Future studies should validate the model in larger and more diverse populations with balanced sex and ethnic distributions and multicenter study designs.

Third, the retrospective, single-center design and inclusion of only patients who underwent both CXR and DXA may have introduced selection and spectrum bias, limiting the generalizability of our findings. In addition, differences in the timing of CXR and DXA examinations may have introduced measurement variability and contributed to AI-DXA disagreement.

Fourth, anatomic non-concordance between the AI target region and the DXA reference sites should be considered when interpreting AI-DXA disagreement. The AI model analyzes the T12–L1 vertebral region, whereas osteoporosis in this study was defined using the established DXA reference standard based on the lumbar spine (L1–L4) and femoral neck. Therefore, a proportion of the observed disagreement between AI predictions and DXA classifications may reflect differences in the anatomical sites assessed rather than true algorithmic misclassification. The AI model is not intended to reproduce site-specific DXA measurements at T12–L1, but rather to use the T12–L1 region as a surrogate radiographic marker of skeletal status for osteoporosis risk according to the DXA-based reference standard. Because bone loss may occur at different rates across axial and appendicular skeletal sites, physiological differences between these anatomical regions may further contribute to discordance between AI-predicted and DXA-derived T-scores. Nevertheless, this anatomical non-concordance is an inherent limitation of opportunistic osteoporosis screening using chest radiographs and may contribute to differences between AI-based predictions and DXA-based classification.

Fifth, although clinical information such as age, sex, height, weight, and fracture history was available, these variables were not incorporated into the AI model [21]. Future studies should investigate whether combining radiographic features with clinical risk factors can further improve prediction performance.

Finally, the model primarily relies on posteroanterior chest radiographs, and the use of a single frontal view may limit the available skeletal information. Recent evidence suggests that incorporating lateral views may further improve BMD estimation [17,19]. Moreover, our study cohort may be susceptible to partial verification bias, as DXA was not performed systematically in all patients undergoing chest radiography but was primarily obtained based on clinical suspicion or recognized risk factors. This non-random selection for DXA verification may have influenced the observed diagnostic performance and could lead to an overestimation of the model’s performance compared with that in an unselected population. Furthermore, optimization of the classification threshold within the validation cohort may have resulted in optimistic estimates of diagnostic performance. External validation in independent cohorts is therefore warranted.

The prevalence of osteoporosis in our hospital-based cohort was 51.2% (1197/2336), which was higher than the reported prevalence in the general population (approximately 22–41%). Because positive and negative predictive values are inherently dependent on disease prevalence, the PPV and NPV observed in this study may not directly generalize to community-based or lower-prevalence opportunistic screening settings. This prevalence effect may also partly explain the lower PPV observed in males (60.00%), who had a lower prevalence of osteoporosis than females in our cohort. Future prospective and multicenter studies are needed to further evaluate the generalizability and clinical utility of the model across populations with different disease prevalences.

## 5. Conclusions

In summary, this independent validation on a male-predominant cohort confirmed the AI model’s high specificity but revealed moderate sensitivity compared to the female-centric pivotal study [17]. The observed performance gap underscores the challenge of demographic domain shifts, particularly the influence of sex-specific bone characteristics in a male population. Consequently, while the model is currently limited as a standalone tool, its high specificity supports its utility for opportunistic risk stratification and DXA prioritization. Future optimization must focus on retraining with diverse datasets to ensure robustness across biological sexes.

## Figures and Tables

**Figure 1:**
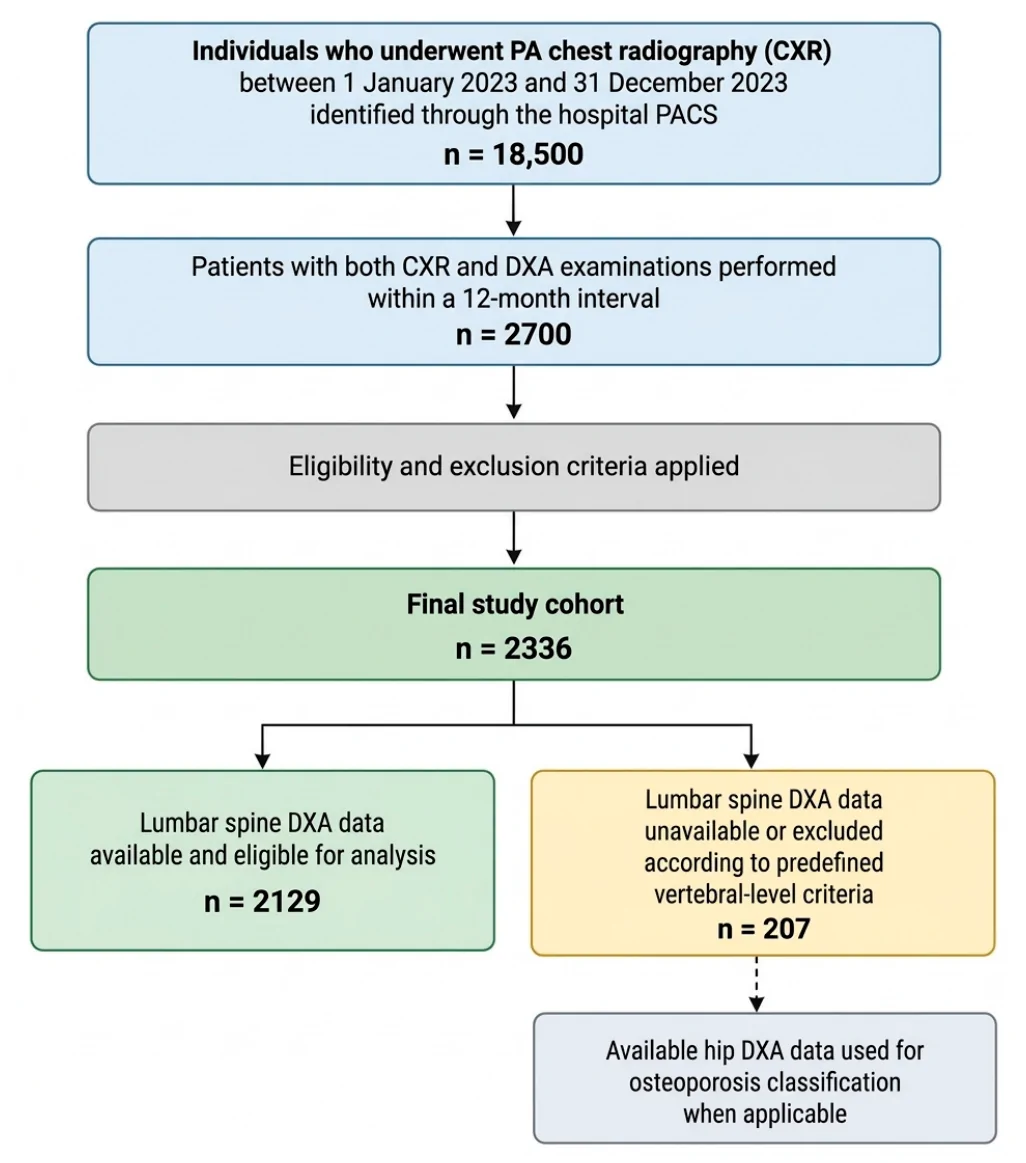
Flowchart of patient selection. Of 18,500 individuals undergoing CXR, 2336 met the predefined eligibility criteria and were included in the final cohort. Lumbar spine DXA data were available in 2129 patients; 207 patients had unavailable or excluded lumbar spine data.

**Figure 2:**
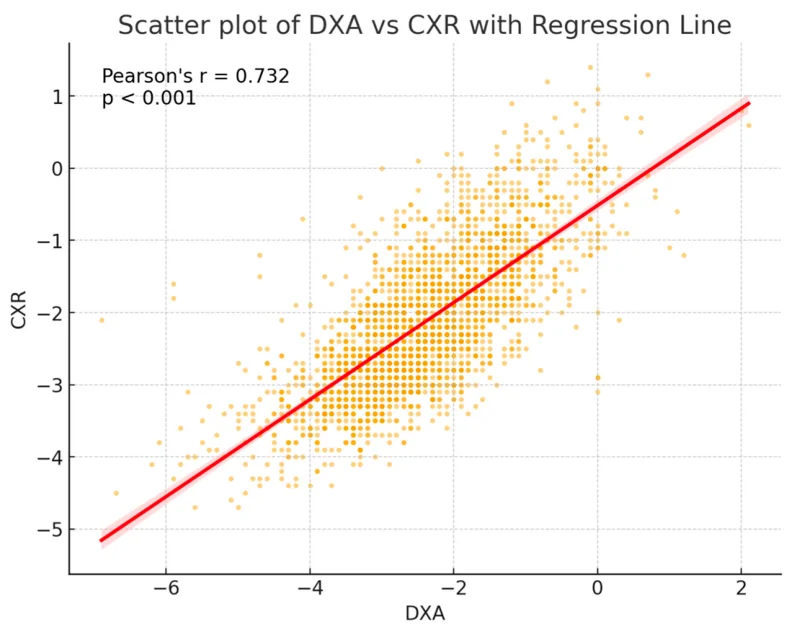
Scatter plot illustrating the correlation between AI-predicted T-scores from chest radiographs (CXR) and the reference T-scores derived from dual-energy X-ray absorptiometry (DXA). The linear regression line (red) and the 95% confidence interval (shaded area) show a substantial positive linear correlation (Pearson’s r = 0.732, *p* < 0.001).

**Figure 3:**
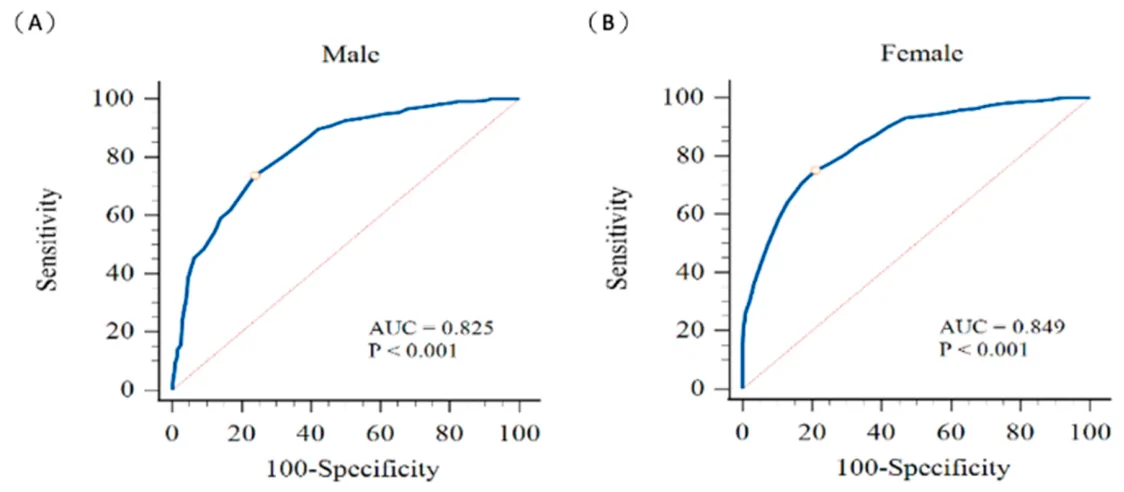
Sex-stratified Receiver Operating Characteristic (ROC) curves demonstrating the diagnostic performance of the deep learning model for detecting osteoporosis. (**A**) Male patients (AUC = 0.825) and (**B**) female patients (AUC = 0.849). The blue curves represent the ROC curves, while the red diagonal lines represent the reference line indicating no discriminative ability (AUC = 0.5).

**Table 1:** Demographic Characteristics.

	Total (*n* = 2336)
Age, years	
Mean ± SD	69.9 ± 10.4
Median (IQR)	70.0 (62.0–78.0)
Sex, *n* (%)	
Male	1688 (72.3%)
Female	648 (27.7%)
Height, cm	
Mean ± SD	157.6 ± 7.8
Weight, kg	
Mean ± SD	59.4 ± 11.2
BMI, kg/m^2^	
Mean ± SD	23.9 ± 3.8
DXA T-score (lowest)	
Mean ± SD	−2.4 ± 1.1
DXA T-score (Spine AP) *n* = 2129	
Mean ± SD	−1.3 ± 1.8

Note: The lowest valid T-score from the lumbar spine or hip measurements was used for the primary osteoporosis classification. Individual lumbar vertebrae with a >1.0 SD T-score difference between adjacent vertebrae were excluded from the lumbar spine calculation. Lumbar spine measurements were unavailable or excluded in 207 patients, who remained in the final cohort; the spine AP T-score was available in 2129 patients for supplementary analysis.

**Table 2:** Diagnostic performance and confusion matrix of VeriOsteo™ OP for osteoporosis detection (T-score ≤ −2.5).

**Part A. 2 × 2 Confusion Matrix**			
**AI Prediction (CXR)**	**DXA Osteoporosis (+)**	**DXA Non-Osteoporosis (−)**	**Total**
AI (+) (Osteoporosis)	834	175	1009
AI (−) (Non-Osteoporosis)	363	964	1327
Total	1197	1139	2336
**Part B. Diagnostic Performance Metrics**			
**Metric**		**Value (95% CI)**	
Sensitivity		69.67% (67.01–72.21)	
Specificity		84.64% (82.43–86.61)	
Positive Predictive Value (PPV)		82.66% (80.20–84.47)	
Negative Predictive Value (NPV)		72.65% (70.18–74.98)	
Accuracy		76.97% (75.22–78.63)	
Area Under the Curve (AUC)		0.854 (0.839–0.868)	

Note: DXA = Dual-energy X-ray Absorptiometry; CXR = Chest X-ray; 95% Confidence Intervals were calculated using the Wilson score method.

**Table 3:** Sex-specific diagnostic performance of the AI model for osteoporosis detection.

Subgroup	Overall	Male	Female
Participants, *n*	2336	648	1688
Osteoporosis prevalence based on DXA, *n* (%)	1197 (51.24%)	212 (32.72%)	985 (58.35%)
AUC (95% CI)		0.825 (0.794–0.854)	0.849 (0.831–0.866)
*p*-value for sex AUC comparison (DeLong’s test)		<0.001	
Sex-Specific AI Cutoff (T-score)		≤−1.9	≤−2.5
Sensitivity (95% CI)		73.58 (67.27–79.06)	74.92 (72.12–77.53)
Specificity (95% CI)		76.15 (71.93–79.91)	79.09 (75.93–81.93)
Accuracy (95% CI)		75.31 (71.85–78.47)	76.66 (74.58–78.61)
PPV (95% CI)		60 (53.94–65.77)	83.39 (80.79–85.70)
NPV (95% CI)		85.57 (81.72–88.72)	69.24 (65.96–72.33)

Notes: Osteoporosis diagnosis was defined using DXA T-score ≤ −2.5 for both sexes; sex-specific AI cutoffs were derived from ROC curve analysis (Youden index) and evaluated in the same cohort; therefore, the corresponding performance estimates are exploratory and require validation in independent cohorts. Values are presented as percentages, with 95% confidence intervals calculated using the Wilson score method. Abbreviations: AUC = area under the receiver operating characteristic curve; CI = confidence interval; PPV = positive predictive value; NPV = negative predictive value; DXA = dual-energy X-ray absorptiometry; AI = artificial intelligence.

## Data Availability

The de-identified dataset supporting the main findings of this study is not publicly available due to institutional privacy and ethical restrictions governing patient-level clinical and imaging data. However, a minimal de-identified dataset containing the variables necessary to reproduce the main analyses may be provided by the corresponding author upon reasonable request, subject to institutional data-sharing requirements and appropriate approval.
